# Coordinated Sampling of Microorganisms Over Freshwater and Saltwater Environments Using an Unmanned Surface Vehicle (USV) and a Small Unmanned Aircraft System (sUAS)

**DOI:** 10.3389/fmicb.2018.01668

**Published:** 2018-08-15

**Authors:** Craig W. Powers, Regina Hanlon, Hinrich Grothe, Aaron J. Prussin, Linsey C. Marr, David G. Schmale

**Affiliations:** ^1^Department of Civil and Environmental Engineering, Virginia Tech, Blacksburg, VA, United States; ^2^Department of Plant Pathology, Physiology, and Weed Science, Virginia Tech, Blacksburg, VA, United States; ^3^Institute of Materials Chemistry, Technische Universität Wien, Vienna, Austria

**Keywords:** aerosol, bioaerosol, ice nucleation, particulates, *Pseudomonas*, unmanned vehicle, 3D-printing, impinger

## Abstract

Biological aerosols (bioaerosols) are ubiquitous in terrestrial and aquatic environments and may influence cloud formation and precipitation processes. Little is known about the aerosolization and transport of bioaerosols from aquatic environments. We designed and deployed a bioaerosol-sampling system onboard an unmanned surface vehicle (USV; a remotely operated boat) to collect microbes and monitor particle sizes in the atmosphere above a salt pond in Falmouth, MA, United States and a freshwater lake in Dublin, VA, United States. The bioaerosol-sampling system included a series of 3D-printed impingers, two different optical particle counters, and a weather station. A small unmanned aircraft system (sUAS; a remotely operated airplane) was used in a coordinated effort with the USV to collect microorganisms on agar media 50 m above the surface of the water. Samples from the USV and sUAS were cultured on selective media to estimate concentrations of culturable microorganisms (bacteria and fungi). Concentrations of microbes from the sUAS ranged from 6 to 9 CFU/m^3^ over saltwater, and 12 to 16 CFU/m^3^ over freshwater (over 10-min sampling intervals) at 50 m above ground level (AGL). Concentrations from the USV ranged from 0 (LOD) to 42,411 CFU/m^3^ over saltwater, and 0 (LOD) to 56,809 CFU/m^3^ over freshwater (over 30-min sampling intervals) in air near the water surface. Particle concentrations recorded onboard the USV ranged from 0 (LOD) to 288 μg/m^3^ for PM1, 1 to 290 μg/m^3^ for PM2.5, and 1 to 290 μg/m^3^ for PM10. A general trend of increasing concentration with an increase in particle size was recorded by each sensor. Through laboratory testing, the collection efficiency of the 3D-printed impingers was determined to be 75% for 1 μm beads and 99% for 3 μm beads. Additional laboratory tests were conducted to determine the accuracy of the miniaturized optical particle counters used onboard the USV. Future work aims to understand the distribution of bioaerosols above aquatic environments and their potential association with cloud formation and precipitation processes.

## Introduction

Aerosols are microscopic particulate matter (PM) that become airborne at the planetary surface and remain suspended in the atmosphere (Vincent, 2007; Millner, 2009). These aerosols can be from anthropogenic or natural sources, i.e., dust and smoke, or can be formed in the atmosphere as secondary aerosols from chemical reactions involving gasses (e.g., sulfur oxides, nitrogen oxides, and volatile organic compounds) (Colbeck, 2014). Transport of some aerosols is known to occur over long distances in the atmosphere (Brown and Hovmøller, 2002; Kellogg and Griffin, 2006; Griffin, 2007; Weil et al., 2017). African dust has been observed to be transported westward over the Atlantic to North America and northward over the Mediterranean to Europe (Creamean et al., 2013). Aerosols can have harmful health effects on humans, and can serve as a central component to environmental problems, for example, photochemical smog, poor air quality, and global warming (Colbeck, 2014). Aerosols can also be biological in nature, and these are often referred to as biological aerosols (bioaerosols) (Després et al., 2012).

Bioaerosols are generally small (about 0.02 to 100 μm), and include bacteria, viruses, fungal spores, pollen, and algae. They can be living or dead cells (Després et al., 2012), and they can also include macromolecules released from cells (Pummer et al., 2012). Some may be highly infectious, can produce hazardous byproducts, or can trigger an immunological response in humans (Cherrie et al., 2011). Some of these bioaerosols, such as the bacterium *Pseudomonas syringae*, are known plant pathogens (Monteil et al., 2016) and have been suggested as contributors to cloud ice nucleation and precipitation events (Morris et al., 2008, 2014; Hallar et al., 2012). This is possible due to the expression of an ice nucleation active (INA) protein allowing *P. syringae* to initiate the freezing of water at temperatures at approximately -2°C, which is much warmer than normally required for water that is free from particulates (Maki et al., 1974; Morris et al., 2008). *P. syringae* is considered to be one of the most effective ice nucleators, biotic or abiotic, and therefore, one of the largest causes of surface frost damage in plants (Maki et al., 1974; Lindow, 1983). Ice-nucleating strains of *P. syringae* have been found in virtually all components of the water cycle, including rain, snow, clouds, and lakes (Morris et al., 2008). Some fungi in the genera *Fusarium* and *Mortierella* have also been reported as ice nucleators (Pouleur et al., 1992; Fröhlich-Nowoisky et al., 2015), but the composition of their ice nuclei have not yet been described in detail. Little is known about the sources, aerosolization, and transport of *P. syringae* and other bioaerosols and their interactions with the environment (Pietsch et al., 2015, 2017; Fröhlich-Nowoisky et al., 2016). New scientific tools are needed to study these bioaerosols and their global impact (Coluzza et al., 2017).

New and improved environmental sensors are enabling researchers to study bioaerosols in natural environments with an unprecedented level of sophistication and detail. Many of these sensors can be mounted on unmanned systems, such as unmanned aircraft systems (UASs) and unmanned surface vehicles (USVs). These unmanned systems can help gather data in a safe and cost-effective manner that, in some cases, would otherwise be impossible for human based endeavors. Coordinated sampling efforts with unmanned systems such as USVs and UASs can be used to study aerosols on over a variety of temporal and spatial scales.

The ultimate goal of this research was to develop a method to monitor bioaerosols above saltwater and freshwater aquatic environments using environmental sensors onboard a USV and a small unmanned aerial system (sUAS). Since *P. syringae* has been found in virtually all components of the water cycle (Morris et al., 2008), we hypothesized that microorganisms are ubiquitous in the air directly above freshwater and saltwater environments. The specific objectives were to (1) design an automated sampler to collect microbes and monitor particle sizes and (2) use a USV and sUAS in a coordinated study to monitor the distribution of microorganisms above a saltwater and a freshwater aquatic environment.

## Materials and Methods

### Study Sites

**FIGURE 1:**
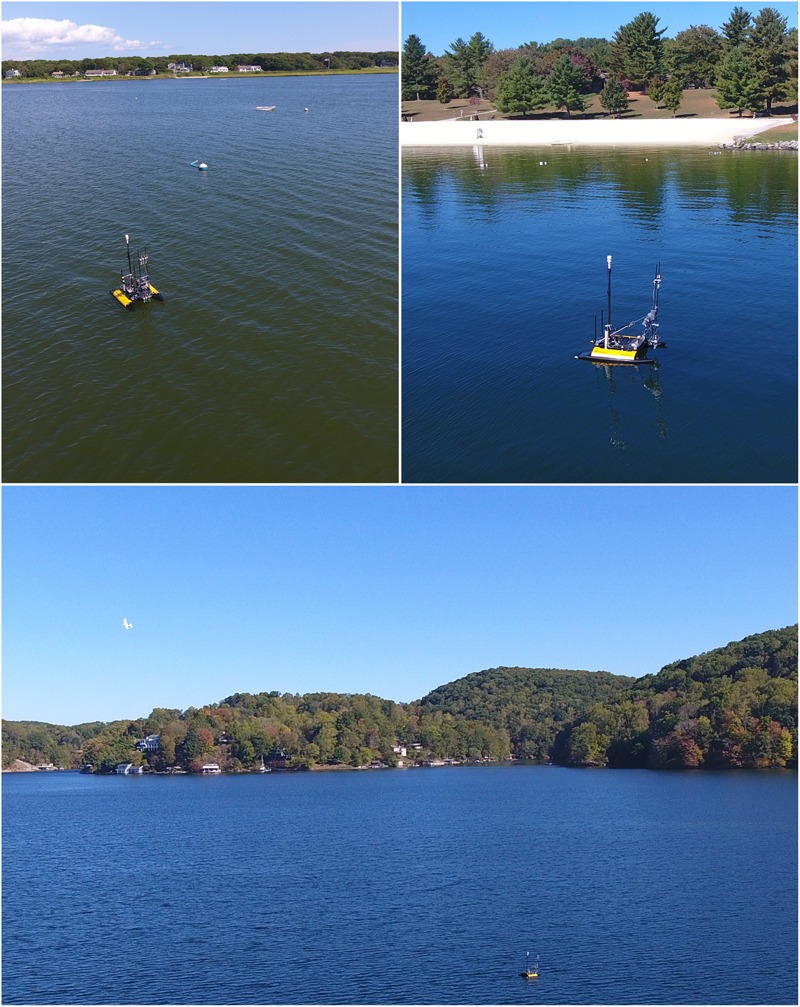
Study sites for sampling bioaerosols with an unmanned surface vehicle (USV) in saltwater (**Top left**, Great Pond, Falmouth, MA, United States) and freshwater (**Top right**, Claytor Lake, Dublin, VA, United States) environments. A small unmanned aircraft system (sUAS) was used to collect samples 50 m above the water (**Bottom**, top left of image), and these flights were coordinated within the USV sampling periods such that both vehicles were sampling simultaneously.

Field experiments were conducted at a saltwater pond, the Great Pond, Falmouth, MA, United States (41.5580N 70.5841W) (**Figure 1**, top left), and a freshwater lake, Claytor Lake, Dublin, VA, United States (37.0530N 80.6208W) (**Figure 1**, top right and bottom). The Great Pond is a large salt water pond that is connected to the ocean. Claytor Lake is a freshwater reservoir fed by the New River.

### Unmanned Surface Vehicle

An electric Kingfisher USV (Clearpath Robotics, Kitchener, ON, Canada) served as the base sampling platform for collecting and measuring bioaerosols in both aquatic environments. This Kingfisher USV has a catamaran hull and was approximately 30 kg in weight with payload, with dimensions 1.35 m × 0.98 m × 1.2 m (L × W × H), as equipped for use in this study. The Kingfisher was operated via remote control. Batteries provided about 3 h of runtime and could be exchanged when needed. The propulsion system was a differential thrust system driven by two impellers inside each of the two hulls giving a maximum speed of 1.7 m/s, allowing for precise movements in shallow waters with little disturbance to the surrounding aquatic environment that could affect sampling. Six 30-min USV sampling missions were conducted at Great Pond, and six 30-min USV sampling missions were conducted at Claytor Lake. For the Great Pond experiments, sampling missions were conducted from about 10:00 AM to 15:00 PM EST on August 20, 2017 (**Table 1**). For the Claytor Lake experiments, sampling missions occurred from about 11:30 AM to 15:30 PM EST on October 3, 2017 (**Table 1**). Each sampling mission consisted of loading six impingers with autoclaved and 0.2 μm filtered media, alternating R2 broth, or R2B (R2 Broth Media 3.15 g/L, Teknova Cat. No. R0005, Hollister, CA, United States), and MO broth, or MOB [Modified Ocean Broth Media 18 g/L Instant Ocean (Spectrum Brands, Blacksburg, VA, United States), 1 μM final NH_4_Cl, 0.1 μM final KH_2_PO_4_] (Reasoner and Geldreich, 1985; Rappé et al., 2002; Joint et al., 2010). The USV then transitioned to the sampling location using visual markers such as buoys for guidance. Once in place, the USV held position using thrusters to stay pointed into the wind and on location. The sampling platform was extended to the vertical position. Data collection from particle and meteorological sensors was started. Vacuum pumps for the appropriate impingers were turned on. Sampling at a rate of 1.6–2.4 LPM was continued for 30 min. At the conclusion of the 30-min sampling duration, the vacuum pumps were turned off and recording of particle and meteorological data was completed. The sampler platform was returned to the stowed configuration and the USV transitioned back to the shore. Impingers were removed, and the 50 mL conical tubes were unscrewed from the impinger body and capped with sterile lids. The impinged samples were then placed on ice and transported to the laboratory for processing after the conclusion of all sampling missions for the day.

**Table 1:** Colony forming unit (CFU; from combined heights of 0.1 and 1 m) and PM data from USV missions at Great Pond (Falmouth, MA, United States) and Claytor Lake (Dublin, VA, United States).

Sample	Date	Time start	Location	Media	CFU/m^3^	PM1 (μg/m^3^)	PM2.5 (μg/m^3^)	PM10 (μg/m^3^)
number		sampling (EST)	(GPS)	type		Min–Max	Min–Max	Min–Max
MA_1	8/20/17	10:15 AM	41.557817, -70.582646	MOB	0	2–8	3–24.1	3–25.4
MA_1	8/20/17	10:15 AM	41.557817, -70.582647	R2B	27			
MA_2	8/20/17	11:09 AM	41.557817, -70.582648	MOB	0	3–5	4.9–7	5.2–8
MA_2	8/20/17	11:09 AM	41.557817, -70.582649	R2B	0			
MA_3	8/20/17	12:28 PM	41.557817, -70.582650	MOB	0	3–4	3–6	3–7
MA_3	8/20/17	12:28 PM	41.557817, -70.582651	R2B	0			
MA_4	8/20/17	12:49 PM	41.557817, -70.582652	MOB	974	4–6	4.6–7	4.9–8
MA_4	8/20/17	12:49 PM	41.557817, -70.582653	R2B	42,411			
MA_5	8/20/17	01:38 PM	41.557817, -70.582654	MOB	96	2–5	3–7	3–10
MA_5	8/20/17	01:38 PM	41.557817, -70.582655	R2B	0			
MA_6	8/20/17	02:25 PM	41.557817, -70.582656	MOB	0	3–5	4–9	4–10
MA_6	8/20/17	02:25 PM	41.557817, -70.582657	R2B	177			
CL_1	10/03/17	11:26 AM	37.052780, -80.619517	MOB	0	4–6	4.1–7	4.9–7
CL_1	10/03/17	11:26 AM	37.052780, -80.619518	R2B	56,809			
CL_2	10/03/17	12:12 PM	37.052780, -80.619519	MOB	0	5–67	4–6	6–7
CL_2	10/03/17	12:12 PM	37.052780, -80.619520	R2B	17,825			
CL_3	10/03/17	01:00 PM	37.052780, -80.619521	MOB	0	1–70	4.9–6.3	5.4–7
CL_3	10/03/17	01:00 PM	37.052780, -80.619522	R2B	22,908			
CL_4	10/03/17	01:58 PM	37.052780, -80.619523	MOB	0	1–52	6–18	6–19
CL_4	10/03/17	01:58 PM	37.052780, -80.619524	R2B	10,662			
CL_5	10/03/17	02:52 PM	37.052780, -80.619525	MOB	0	0–288	3–4	3–4
CL_5	10/03/17	02:52 PM	37.052780, -80.619526	R2B	15,416			
CL_6	10/03/17	03:35 PM	37.052780, -80.619527	MOB	0	0–29	2.5–4	2.6–4.4
CL_6	10/03/17	03:35 PM	37.052780, -80.619528	R2B	5,130			

### Small Unmanned Aircraft System (sUAS)

A small unmanned aircraft system (sUAS) was used to collect microorganisms in the lower atmosphere as described by Jimenez-Sanchez et al. (2018). Flights were coordinated within the USV sampling periods such that both vehicles were sampling simultaneously. The sUAS flew an orbital (circular) pattern with a target altitude of 50 m above ground level (AGL), with the USV at the approximate center of the orbit. The sampling device was closed during takeoff and landing, and was opened by remote control from the ground once the sUAS was at the target sampling altitude and airspeed. The device remained open for the duration of the 10-min sampling interval. Immediately following sample collection, the exposed plate containing agar media [alternating R2A (R2 as described above with 15% agar, Thermo Fisher Scientific Cat. No. 9002-18-0, Asheville, NC, United States) and MOA (MO as described above with 5.8 g/L Gelzan gelling agent, PhytoTechnology Laboratories Cat. No. G3251, Lenexa KS 66215)] was removed from the sampling device and stored in a small plastic container for transport to the laboratory. Unexposed (control) plates were placed in the same plastic container, and incubated under the same conditions as sUAS samples. Flights were conducted by a Federal Aviation Administration (FAA)-certified pilot (Schmale) under Remote Pilot Certificate Number 4038906 with an observer (McClelland). Flights over Claytor Lake, Dublin, VA, United States were conducted under Virginia State Parks Special Use Permit Number 4-012-017 issued by Chris Doss, Park Manager.

### Development of an Automated Atmospheric Sampler and Sensor Integration on a USV

**FIGURE 2:**
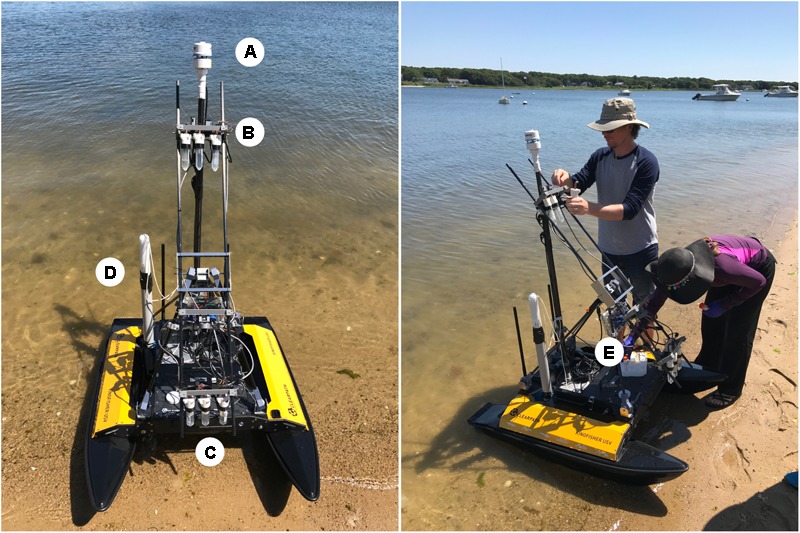
Schematic of atmospheric sampling devices deployed on the USV at Great Pond, Falmouth, MA, United States. An Airmar 200WX sensor **(A)** was used to capture meteorological data. A set of three impingers and two particle counters were used at 1.1 m **(B)** and 0.1 m **(C)** when deployed. A Turner turbidity sensor **(D)** was also used. The sampler is seen in the stowed configuration **(E)**, and was extended to a vertical position when sampling.

**FIGURE 3:**
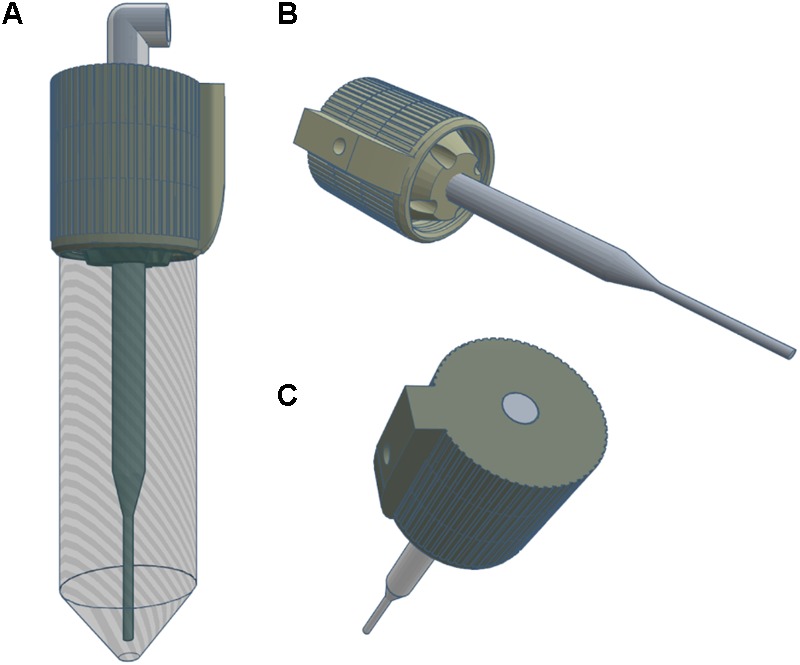
Engineering diagram of the 3D-printed impinger used for the study. The impinger contained a 9.5 mm diameter copper elbow and 50 mL conical tube **(A)**. A borosilicate Pasteur pipette **(B)** was used as a down tube for air flow allowing deposition of microorganisms in the collection fluid. Air entering the inlet at the top of the impinger **(C)** came in contact with only the glass pipette tube and copper elbow minimizing particulate adhesion before deposition.

A bioaerosol-sampling system was integrated into the USV to sample at different heights above the water. The system was designed to extend the water-sampling capabilities of this platform (Powers et al., 2018), though we did not collect water samples as part of the field campaigns described in this manuscript. Four optical particle counters from two different manufactures, the SDS021 (Nova Fitness Co., Ltd., Jinan, Shandong Province, China) and the PMS7003 (Plantower, Shunyi District, Beijing, China), were used (**Figure 2**). One of each type of sensor was placed at 0.1 and 1.1 m above the water, so that at each height, there were two different types of sensors (**Figure 2**). The SDS021 reported PM2.5 and PM10 concentrations while the PMS7003 reported PM1, PM2.5, and PM10 concentrations. These sensors collected measurements at a sampling rate of about 1 Hz. In addition to the PM counters, a custom impinger was designed and printed from high-density polyethylene (which allowed the impinger to be autoclaved) (see Supplementary Files for 3D-printing (.stl) files here: https://github.com/SchmaleLab/Schmale-Lab-3D-Printing-Files-Powers-et-al-Frontiers-2018) (**Figure 3**). The impinger was designed to be screwed directly onto a 50 mL sterile polypropylene conical centrifuge tube that served as the collection vessel (Thermo Fisher Scientific Cat. No. 05-538-60, Asheville, NC, United States). The impinger was also designed around disposable borosilicate Pasteur pipettes (Corning CLS7095D5X, SIGM-ALDRICH, St. Louis, MO, United States) serving as the down tube that air would travel through into the impinger liquid (**Figure 3**). A 1 cm diameter copper plumbing elbow served as the air inlet to the impinger (**Figure 3**). Lin et al. (1997) tested different impinger designs, which guided the design of the impinger used in this study to ensure the highest possible collection efficiency. Three of these impingers where used in two groups at each height that included two PM counters (one of each manufacturer) with one sensor group at 0.1 m above the water surface [**Figure 2C**, impingers 4 (i4), 5 (i5) and 6 (i6) from left to right] and the second sensor group at 1.1 m above the water surface [**Figure 2B**, impingers 1 (i1), 2 (i2) and 3 (i3) from left to right]. Each impinger was connected to a pump modified to operate as a vacuum pump (ZT370-01, Dongguan Zhentian Precision Electronics Co., Ltd., Dongguan city, Guangdong province, China) A flowmeter was used to determine the sampling rate of each vacuum pump (L/min across three independent testing cycles). Impingers i1 and i6 had a mean rate of 1.0 and 1.03 L/min, respectively; a combined sampling rate of 2.03 L/min was used after i1 and i6 sample volumes were combined (i1 and i6 each contained 20 mL of MOB media). Impingers i3 and i4 had a mean rate of 0.61 and 1.27 L/min, respectively; a combined sampling rate of 1.88 L/min was used after i3 and i4 sample volumes were combined (i3 and i4 each contained 20 mL of R2B media). Air was pulled into the impinger nozzle down the pipette tube where it then traveled through 20 mL of liquid media in the conical tube. Control tubes i2 and i5 contained 20 mL of MOB and R2B, respectively, for even numbered missions and 20 mL of R2B and MOB, respectively, for odd numbered missions. These sensor groups were mounted on a two vertical carbon fiber tubes attached to a carbon fiber tubing base attached to the USV (**Figure 2**). The carbon fiber base allowed the vertical sensor assembly to be extended forward in an arc about 45 degrees aft via a stepper motor screw assembly for safe transport between sampling missions. The sensors, vacuum pumps, and stepper motor where connected to a microcontroller (Teensy 3.6, PJRC.COM, LLC., Sherwood, OR, United States) that controlled all sensor operations. An Airmar 200WX marine weather station was integrated into the USV to capture environmental data including wind speed, wind direction, and temperature. All sensor data was transmitted to the USV computer via serial communications for recording. Sensor actuation and data collection were controlled through the USV computer by a command computer on shore over a 2.4 GHz WiFi data link.

### Collection and Culturing of Microorganisms From sUAS

Agar collection plates from the sUAS sampling missions contained R2A or MOA. R2A medium was used to favor the growth of freshwater bacteria, and MOA medium was used to favor the growth of saltwater bacteria. R2A plates were incubated for 3–4 days at room temperature, MOA plates were incubated for 4–10 days at 15°C, and colony forming units (CFUs) were counted and the plates were photographed. A subset of the colonies on each plate were picked with sterile toothpicks and inoculated into 140 μL of H_2_O for ice nucleation assays and subsequent storage at minus 80°C in 20% glycerol. CFU per plate per sUAS flight were converted to CFU/m^3^ of air sampled as per (Aylor et al., 2011; Lin et al., 2014).

### Laboratory Experiments to Determine Sampling Efficiency of the 3D-Printed Impingers and the Accuracy of the Optical Particle Counters

Polystyrene latex (PSL) beads with mean sizes of 3.0 μm (cat #LB30-1ML, Sigma) and 1.0 μm (cat #89904, Sigma), similar in size to bacteria, were chosen to determine impinger collection efficiency and the accuracy of the optical particle counters relative to the APS. A nebulizer, supplied with HEPA filtered air through a hydrocarbon trap, was used to aerosolize the PSL beads. Following aerosolization, the aerosols were routed through a dryer (TSI Model 3062, Shoreview, MN, United States), a Kr-85 neutralizer (TSI, Shoreview, MN, United States), and into a 280L chamber (AtmosBag two-hand, size M, Cat. #Z530212, Sigma-Aldrich, St. Louis, MO, United States). An impinger (described above) containing 30 mL of filter sterilized water (Millipore # GSWP04700), two optical particle counters (SDS021 and PMS7003), and a fan (to promote mixing) were placed inside the chamber. PSL beads were nebulized for 2 min to fill the chamber, and the chamber was allowed to equilibrate for 5 min before sampling. The aerosol size distribution and concentration were determined with an Aerodynamic Particle Sizer (TSI Model 332100, Ser. #71102298, Shoreview, MN, United States) during 2-min sampling runs. This experiment was conducted in triplicate. Collection efficiency (%) was calculated as the [(*C*_atmosbag_ - *C*_impingeroutlet_)/*C*_atmosbag_]×100, where *C* is the particle concentration of the PSL beads.

### Statistical Analyses

Statistical analyses were conducted using R version 3.4.2. A linear regression model was used to examine differences among culturable bacteria collected during 12 sampling missions over 2 days with meteorological and PM concentration data. A circular linear regression model was used to compare wind direction with PM concentration data. A 95% confidence interval was used for significant differences (*P* < 0.05).

## Results

### Missions at Great Pond, Falmouth, MA, United States

**FIGURE 4:**
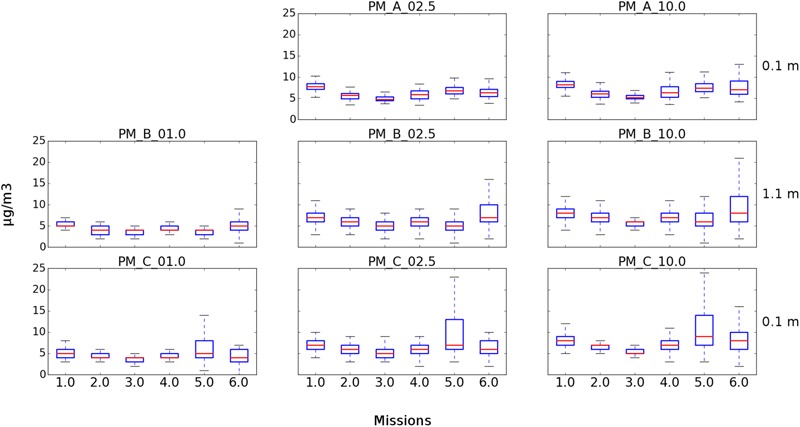
Box plots showing PM concentrations from USV missions at Great Pond, Falmouth, MA, United States. Three of the four integrated particle sensors were operational for the six missions. **(Top)** Represents the SDS021 sensor at 0.1 m for PM2.5 and PM10. **(Middle)** Represents the PM7003 sensor at 1.1 m for PM1, PM2.5, and PM10. **(Bottom)** Represents the PM7003 sensor at 0.1 m for PM1, PM2.5, and PM10.

**FIGURE 5:**
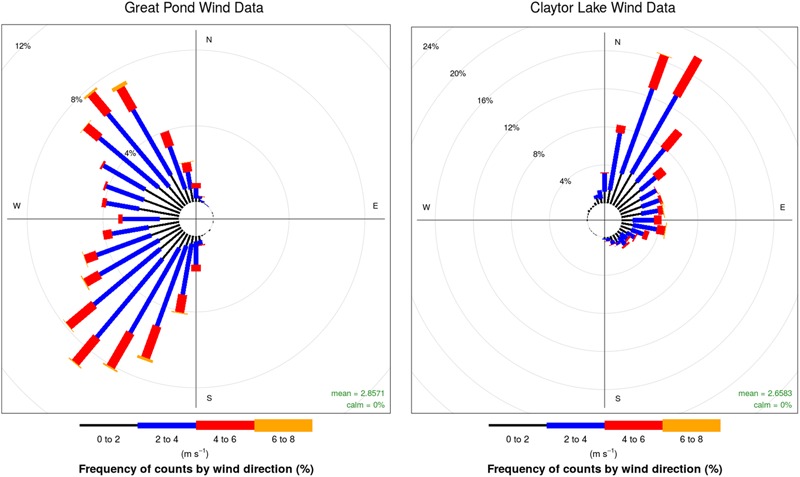
Wind plots for the Great Pond, Falmouth, MA, United States **(Left)** and Claytor Lake, Dublin, VA, United States **(Right)**. *Colors* represent the speed of wind with length of bars representing the percentage of time at the given wind speed.

Six USV missions and four sUAS missions were conducted on August 20, 2017 at the Great Pond, Falmouth, MA, United States (**Tables 1, 2**). The concentration of culturable microorganisms from USV missions ranged from 0 (LOD) to 42,411 CFU/m^3^ on R2A and from 0 (LOD) to 974 CFU/m^3^ on MOA, respectively (**Table 1**). PM concentrations from USV missions ranged from 2 to 8 μg/m^3^ for PM1, 3 to 24.1 μg/m^3^ for PM2.5, and 3 to 25.4 μg/m^3^ for PM10 (**Table 1**). The concentration of culturable microorganisms (bacteria and fungi) from UAS flights ranged from 6 to 9 CFU/m^3^ (over 10-min sampling intervals) (**Table 2**). **Figure 4** shows PM concentrations for the six missions with outliers removed. Unexposed plates of R2A and MOA (controls for sUAS missions) did not yield any culturable microorganisms. Impinger control collections (i2 and i5) combined across missions for matching media types (R2B or MOB) did not yield any culturable microorganisms. Wind speed varied from 1 to 9.5 knots (0.5 to 5.0 m/s) at the Great Pond, Falmouth, MA, United States (**Figure 5**).

**Table 2:** Concentrations of microbes from sUAS missions at Great Pond (FSalt) and Claytor Lake (FFresh).

Flight	Date	Time plate	Location	Media	CFU/plate	CFU/m^3^	Colonies	Ice+	% Ice+
number		open (EST)	(GPS)	type			screened		
FSalt1	8/20/2017	11:16 AM	41.557817, -70.582646	MOA	29	6	12	0	0
FSalt2	8/20/2017	12:05 PM	41.557817, -70.582646	R2A	40	9	18	0	0
FSalt3	8/20/2017	12:52 PM	41.557817, -70.582646	MOA	35	8	18	0	0
FSalt4	8/20/2017	1:43 PM	41.557817, -70.582646	R2A	44	9	16	0	0
FFresh1	10/3/2017	11:44 AM	37.052780, -80.619517	MOA	60	13	13	0	0
FFresh2	10/3/2017	12:24 PM	37.052780, -80.619517	R2A	59	13	24	0	0
FFresh3	10/3/2017	2:06 PM	37.052780, -80.619517	MOA	72	16	24	1	4
FFresh4	10/3/2017	2:59 PM	37.052780, -80.619517	R2A	55	12	25	4	16

### Missions at Claytor Lake, Dublin, VA, United States

**FIGURE 6:**
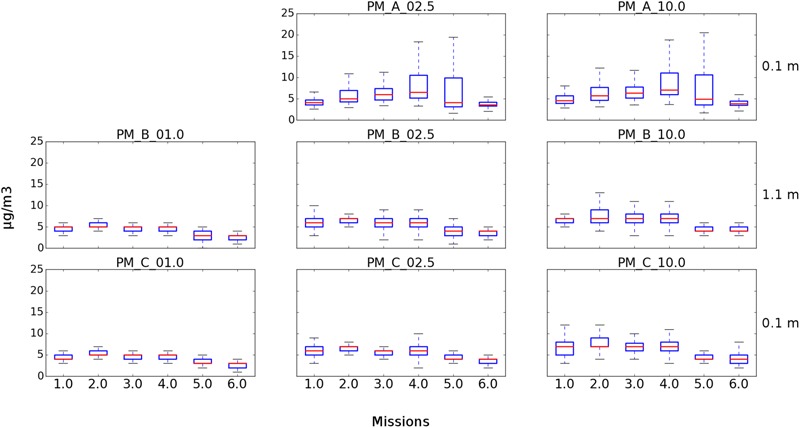
Box plots showing PM concentrations from USV missions at Claytor Lake, Dublin, VA, United States. Three of the four integrated particle sensors were operational for the six missions. **(Top)** Represents the SDS021 sensor at 0.1 m for PM2.5 and PM10. **(Middle)** Represents the PM7003 sensor at 1.1 m for PM1, PM2.5, and PM10. **(Bottom)** Represents the PM7003 sensor at 0.1 m for PM1, PM2.5, and PM10.

Six USV missions and four UAS missions were conducted on October 3, 2017 at Claytor Lake, Dublin, VA, United States (**Tables 1, 2**). The concentration of culturable microorganisms from USV missions ranged from 5,130 to 56,809 CFU/m^3^ on R2A (**Table 1**). No culturable microorganisms were observed on MOB (**Table 1**). PM concentrations from USV missions ranged from 0 (LOD) to 288 μg/m^3^ for PM1, from 2.5 to 18 μg/m^3^ for PM2.5, and from 2.6 to 19 μg/m^3^ for PM10 (**Table 1**). **Figure 6** shows PM concentrations for the six missions (with outliers removed). The concentration of culturable microorganisms (bacteria and fungi) from UAS flights ranged from 12 to 16 CFU/m^3^ (over 10-min sampling intervals) (**Table 2**). Unexposed plates of R2A and MOA (controls for sUAS missions) did not yield any culturable microorganisms. Impinger control collections (i2 and i5) combined across missions for matching media types (R2B or MOB) did not yield any culturable microorganisms. Wind speed varied from 1.4 to 9.5 (0.7 to 5.0) knots during sampling at Claytor Lake, Dublin, VA, United States (**Figure 5**).

### Laboratory Experiments to Determine Sampling Efficiency of the 3D-Printed Impingers

For the 1 μm beads, the peak of the aerosol size distribution in the chamber was 1.11 μm, matching the nominal size of the beads well. At this size, the impinger efficiency was 75.27 ± 8.26% (data not shown). For the 3 μm beads, the peak of the aerosol size distribution was 3.28 μm, matching the nominal size of the beads well. At this size, the impinger efficiency was 99.32 ± 0.590% (data not shown).

**FIGURE 7:**
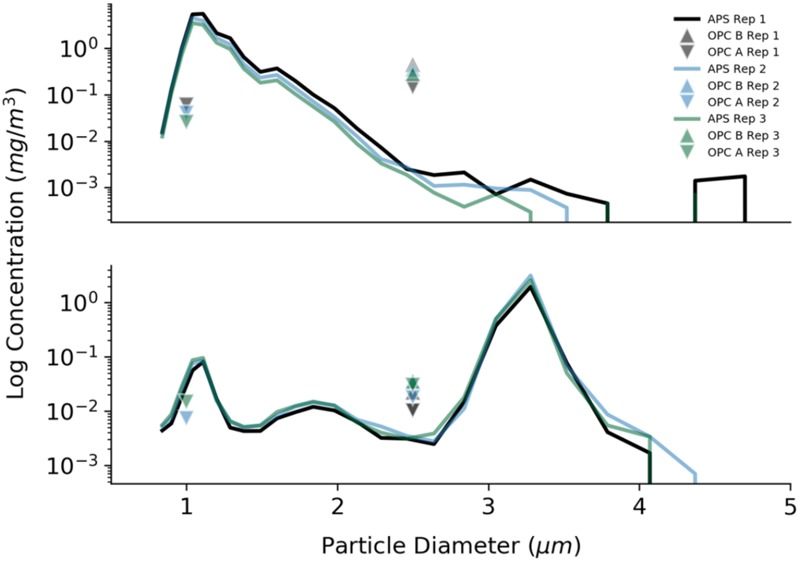
Laboratory tests to determine the accuracy of the optical particle counters used in this study. Results are presented for three repetitions of two different trials. The log of the particle concentration for 1 μm beads (trial 1, **Top**) and 3 μm beads (trial 5, **Bottom**) from the APS is compared to the readings from the two different optical particle counters (OPCA and OPCB).

Comparisons between the reported concentration for the PMS7003 OPC (OPCA) and SDS021 OPC (OPCB) sensors to the TSI APS during trials are shown in **Figure 7**. High particle concentrations during laboratory testing may have contributed to observed differences in concentrations reported by the PM sensors compared to those reported by the APS.

### Results of Statistical Analyses

The SDS0021 sensor at 0.1 m showed a significant correlation with wind speed reported by the USV mounted meteorological sensor at Claytor Lake, Dublin, VA, United States for PM2.5 (*P* < 0.001), PM2.5 and PM10 (*P* < 0.001). Correlations were also found at the Great Pond, Falmouth, MA, United States for wind speed with PM2.5 (*P* = 0.016) and PM10 (*P* = 0.006) for the PMS7003 senor at 0.1 m and PM2.5 (*P* < 0.001) and PM10 (*P* = 0.0316) for the SDS0021 sensor at 0.1 m. PM sensors for Claytor Lake, Dublin, VA, United States reported similar concentrations (*P*-values were <0.001), except the SDS0021 sensor at 0.1 m with both PMS7003 sensors at 0.1 and 1.1 m for PM2.5 and PM10 (*P-*values ranged from 0.299 to 0.551). For Great Pond, Falmouth, MA, United States concentrations were on average higher at 1.1 than 0.1 m for both of the PM sensors (OPCA and OPCB) across all PM sizes reported. Both PM sensors for the Great Pond, Falmouth, MA, United States reported similar concentrations (*P*-values were <0.001). For Claytor Lake, Dublin, VA, United States concentrations were on average lower at 1.1 than 0.1 m for both of the PM sensors (OPCA and OPCB) across all PM sizes reported. PM concentrations were highly correlated with wind direction for all sensors and PM sizes at Claytor Lake, Dublin, VA, United States (*P <* 0.001) and the Great Pond, Falmouth, MA, United States (*P*-values were <0.001).

## Discussion

The sources, distribution, and transport of bioaerosols are not well understood. New information is needed regarding the impacts of bioaerosols on climate (Morris et al., 2014; Fröhlich-Nowoisky et al., 2016). We developed and implemented a bioaerosol-sampling system onboard a USV to collect and characterize bioaerosols at multiple heights above saltwater and freshwater environments. The main focus of this research was the development and demonstration of new technologies with unmanned systems to collect and characterize bioaerosols in aquatic environments. In this pilot project, we demonstrated that unmanned systems operating in the air and the water could be used in a coordinated fashion to explore atmospheric processes.

Several sensors were integrated into our bioaerosol-sampling system, including a series of 3D-printed impingers, two different brands of optical particle sizers (SDS021 and PMS7003), and a meteorological sensor. A sUAS was used to collect microorganisms on agar media 50 m above the surface of the water. Use of coordinated unmanned systems with atmospheric samplers and sensors can be used to characterize the distribution and transport of microbes and aerosols above aquatic environments. Concentrations from the USV ranged from 0 (LOD) to 42,411 CFU/m^3^ over saltwater, and 0 (LOD) to 56,809 CFU/m^3^ over freshwater (over 30-min sampling intervals) in air near the water surface. Mayol et al. (2014) reported airborne concentrations of prokaryotes between 2,782 and 19,132 cells/m^3^ over the North Atlantic Ocean, with an average of about 8,000 cells/m^3^. These observations represent an extremely wide range of concentrations, and may point to the temporal and spatial variability of bioaerosols in aquatic environments. Some microorganisms such as *P. syringae* are ubiquitous in aquatic environments (Morris et al., 2008), and have been found to be highly variable in spatial distribution in freshwater lakes (Pietsch et al., 2017). This variability could have an impact on the large range of concentrations of culturable bacteria. Though was no significant difference in bacterial concentrations collected on the USV on both R2A and MOA media for each mission (P-values ranged from *P* = 0.17 to *P* = 0.92), the magnitude of concentrations between the Great Pond and Claytor Lake was noteworthy and likely due to the different aquatic environments and the R2A and MOA media used to simulate those environments. The lack of correlation from PM sensors could be due to the lack of accuracy of the units and/or due to the small sample size, at least in part. Previous investigations of relationships between PM sensors and atmospheric concentrations of microbes have shown that these associations are complicated and are never straightforward (e.g., Sousa et al., 2008; Raisi et al., 2013; Zhai et al., 2018). Moreover, the viable (culturable) portion of airborne microorganisms is typically below 10% (Burrows et al., 2009). If only a small fraction of atmospheric microbes is actually detectable via culturing, and if this fraction changes independently from the aerosolized volume of bioaerosols (i.e., due to environmental factors), this could mask correlations between bioaerosols and PM. Additional efforts to synchronize near real-time measurements across additional sampling times and seasons are warranted (e.g., sampling during the night and during winter months with shorter sampling intervals, such that the impingers more closely overlap with the data from the optical particle counters), along with considering total cell counts (in addition to CFUs) in samples using methods such as epifluorescence and flow cytometry.

**FIGURE 8:**
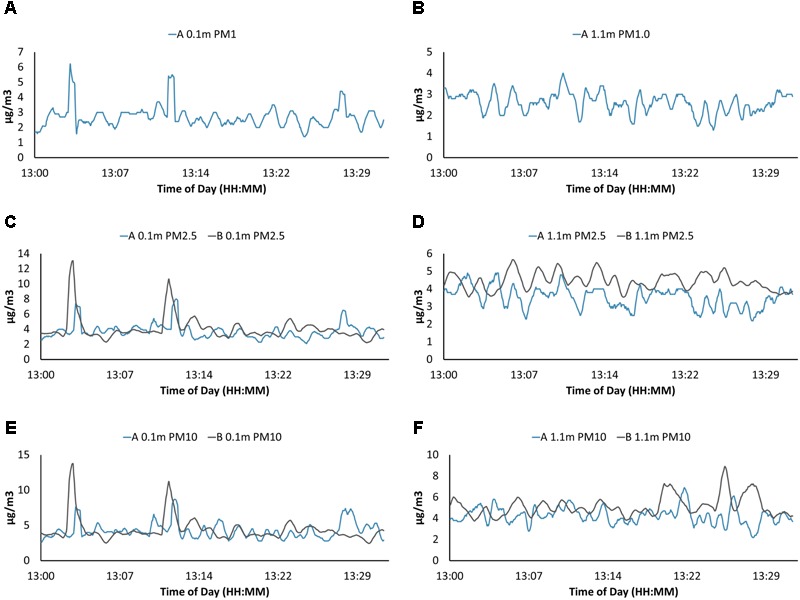
Particulate matter (PM) data from both PM sensors for USV missions on Claytor Lake, Dublin, VA, United States on October 3, 2017. Two models of optical particle counters PMS7003 **(A)** and SDS021 **(B)** were used. Only the PMS7003 **(A)** sensor was able to resolve PM1 at 0.1 m above the water surface **(A)** and PM1 at 1.1 m **(B)**. Both models reported PM2.5 at 0.1 m **(C)**, PM2.5 at 1.1 m **(D)**, PM10 at 0.1 m **(E)**, and PM10 at 1.1 m **(F)**. Graphs are in general accordance both in shape and averages with some minor variations.

Wind speed varied from 1 to 9.5 knots (0.5 to 5.0 m/s) at the Great Pond, Falmouth, MA, United States and varied from 1.4 to 9.5 (0.7 to 5.0) knots during sampling at Claytor Lake, Dublin, VA, United States (**Figure 5**). PM concentrations from the four PM sensors were in general agreement in average concentrations and concentration variations (**Figure 8**). Particle sensors at 0.1 m showed a significant correlation with wind speed reported by the USV mounted meteorological sensor at Claytor Lake, Dublin, VA, United States and at the Great Pond, Falmouth, MA, United States. The connection with higher particle concentrations with increased wind suggests vertical mixing at the MABL where the characteristic timescale for mixing of gasses and aerosols is on the order of seconds (Jonsson et al., 2014). This connection is what we should expect to see and is partial validation of the sensors ability to operate effectively in this time scale at this altitude. Different levels of sensitivity or accuracy between the PMS7003 and SDS0021 sensors could explain the consistently different correlation of the sensors. The lack of correlation at the 1 μm particle size could indicate a continuous background noise of sea salt particulates being reported at the MABL (Gong et al., 1997).

Flights with the sUAS were conducted at the same target altitude across missions (50 m AGL), so that the microbes cultured from these missions could be compared across sUAS flights conducted at a similar altitude (e.g., Tallapragada et al., 2011; Lin et al., 2014). Future investigations should include profiles of the atmosphere directly above the aquatic environments (or on land adjacent to the water) using portable SODAR instruments (Finn et al., 2017) and/or hovering rotary wing aircraft equipped with wind monitors such as sonic anemometers (Palomaki et al., 2017). Such efforts could help orchestrate adaptive sampling missions with unmanned systems, so they can target specific layers of the atmosphere. Moreover, our impinger system could be integrated into a hovering quadcopter in the future, enabling the same sampling technology across different platforms in the air and in water (as opposed to the present study, where we compare CFUs collected on Petri plates on the sUAS to CFUs recovered from the impingers onboard the USV).

To collect bioaerosols, researchers typically either use a filtration or impingement method (Duchaine et al., 2001). Liquid impingement is preferred when bioaerosol viability needs to be preserved, as some filters can cause bacteria to desiccate and lose viability (Jensen et al., 1992). Commercial impingers (e.g., BioSampler, All Glass Impinger) have a high collection efficiency and preserve microbial viability, but they are fairly expensive. To our knowledge, this is the first published report that used inexpensive 3D-printed impingers (less than $1/impinger) to collect bioaerosols. The 3D-printed impingers used in this study exhibited a very high collection efficiency (>99 and >75% for 3 and 1 μm particles, respectively). Also, due to the low cost of the 3D-printed impinger, multiple samples could be collected in parallel. Further, the design allows a single sample to be collected and then sealed until analysis (e.g., the liquid does not need to be transferred into another vesicle after sampling, followed by disinfestation of the impinger prior to collecting another sample) which significantly reduces the potential for contamination. Citizen science is an emerging field that uses the community to collect data and answer research questions that require large datasets (Bonney et al., 2009; Dunn et al., 2013). The 3D-printed impingers used in this study have great potential for use in the citizen science community, as studying bioaerosols has remained fairly elusive in the citizen science realm in part due to the traditionally high costs associated with air sampling equipment.

Sea salt aerosols are important as they play significant roles in clear sky radiative forcing and serve as a source of cloud condensation nuclei (Winter and Chýlek, 1997; Gras and Keywood, 2017). Understanding the components of the aerosols reported by the particle counters, sea salt or otherwise, remains a challenge and will require further testing and validation of all sensors. Future work could probe deeper associations of connections of culturable bacteria, wind speed, and particle concentrations at the air–water interface. Such work could contribute to our understanding of the sources and transport of biotic and abiotic aerosols and their linkages to global health.

## Author Contributions

CP designed, fabricated, and deployed the atmospheric sampling system onboard the USV, served as the Pilot in Command (PIC) for all USV missions, conducted laboratory experiments to determine the sampling efficiency of the impingers and accuracy of the particle monitors, analyzed PM and culture data, and led the writing of the manuscript. RH assisted in all field campaigns, cultured microbes from impinging samples, conducted ice nucleation assays, managed the strain collection, assisted with laboratory experiments to determine the sampling efficiency of the impingers and accuracy of the particle monitors, analyzed data, and assisted in writing the manuscript. HG assisted in the field campaigns in Great Pond, Falmouth, MA, United States and assisted in writing the manuscript. AP assisted with laboratory experiments to determine the sampling efficiency of the impingers and accuracy of the particle monitors, analyzed data, and assisted in writing the manuscript. LM analyzed the data and assisted in writing the manuscript. DS managed the project, designed the experiments, was the PIC for all sUAS missions, analyzed the data, and assisted in writing the manuscript.

## Conflict of Interest Statement

The authors declare that the research was conducted in the absence of any commercial or financial relationships that could be construed as a potential conflict of interest.
